# A New Mouse Line Reporting the Translation of Brain-Derived Neurotrophic Factor Using Green Fluorescent Protein

**DOI:** 10.1523/ENEURO.0462-19.2019

**Published:** 2020-01-09

**Authors:** Erin Wosnitzka, Xinsheng Nan, Jeff Nan, Pedro Chacón-Fernández, Lothar Kussmaul, Michael Schuler, Bastian Hengerer, Yves-Alain Barde

**Affiliations:** 1 School of Biosciences, Cardiff University, Cardiff CF10 3AX, United Kingdom; 2 Boehringer-Ingelheim, Biberach an Der Riss 88397, Germany

**Keywords:** BDNF, GFP, imaging, monoclonal antibodies, transfection, transgenics

## Abstract

While BDNF is receiving considerable attention for its role in synaptic plasticity and in nervous system dysfunction, identifying brain circuits involving BDNF-expressing neurons has been challenging. BDNF levels are very low in most brain areas, except for the large mossy fiber terminals in the hippocampus where BDNF accumulates at readily detectable levels. This report describes the generation of a mouse line allowing the detection of single brain cells synthesizing BDNF. A bicistronic construct encoding BDNF tagged with a P2A sequence preceding GFP allows the translation of BDNF and GFP as separate proteins. Following its validation with transfected cells, this construct was used to replace the endogenous *Bdnf* gene. Viable and fertile homozygote animals were generated, with the GFP signal marking neuronal cell bodies translating the *Bdnf* mRNA. Importantly, the distribution of immunoreactive BDNF remained unchanged, as exemplified by its accumulation in mossy fiber terminals in the transgenic animals. GFP-labeled neurons could be readily visualized in distinct layers in the cerebral cortex where BDNF has been difficult to detect with currently available reagents. In the hippocampal formation, quantification of the GFP signal revealed that <10% of the neurons do not translate the *Bdnf* mRNA at detectable levels, with the highest proportion of strongly labeled neurons found in CA3.

## Visual Abstract

**Figure:**
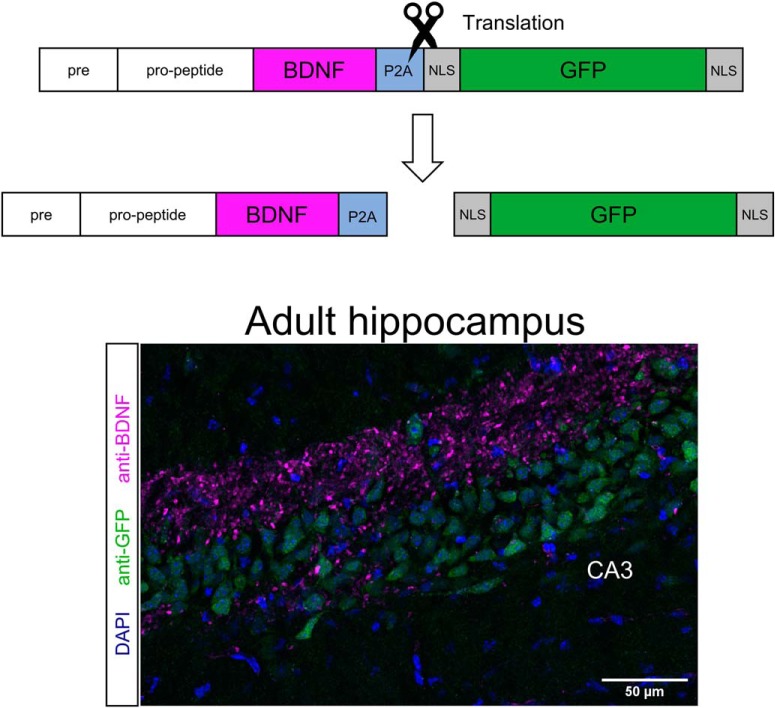

## Significance Statement

BDNF is a highly conserved growth factor known to be essential for the function of the nervous system. Its very low abundance in the brain has retarded the development of drugs targeting BDNF-expressing neurons in disease-relevant brain areas. The present report describes a novel approach allowing the localization of single cells in the adult mouse brain actively translating *Bdnf* mRNAs using GFP as a surrogate marker. The availability of these transgenic animals will also help in understanding the action of drugs such as ketamine that are thought to act by increasing *Bdnf* translation.

## Introduction

Brain-derived neurotrophic factor (BDNF) is a secreted growth factor required for the development and function of the nervous system (Mitre et al., 2017). In humans, decreased levels of BDNF have been associated with a wide range of conditions, including neurodegeneration (Mariga et al., 2017). In addition, there is considerable evidence for a role of BDNF in depression (Castrén and Kojima, 2017) and memory (Egan et al., 2003; Heldt et al., 2007). There are large differences in the levels of *Bdnf* transcription between different brain regions and from one neuron to the next as long documented by *in situ* hybridization studies in the adult brain of mice, rats, and pigs (Hofer et al., 1990; Wetmore et al., 1990). Given that *Bdnf* transcription is regulated by neuronal activity in excitatory neurons (Tao et al., 1998), different degrees of activity most likely contribute to these differences. However, comparisons between the staining intensity of BDNF with surrogate markers of activity such as Arc (Dieni et al., 2012; Nikolaienko et al., 2018) suggest that other determinants are also likely to play a role. To better understand the mechanisms regulating the translation of *BDNF* and to facilitate the development of new drugs targeting BDNF-expressing neurons, it is desirable to use approaches allowing the characterization of single cells as a function of the intensity of a reporter signal such as GFP. Feasibility is suggested by previous work using vectors encoding the regulatory sequences of *Bdnf* to drive the expression of reporters including GFP (Guillemot et al., 2007; Koppel et al., 2009; Fukuchi et al., 2017). In addition, detectable levels of fluorescence have been illustrated using sequences encoding fluorescent proteins inserted within activity-dependent exons of *Bdnf* (Singer et al., 2018). These previous results indicate that the strength of the *Bdnf* promoters drives levels of GFP expression sufficient to allow single-cell visualization and sorting. Here we report on the substitution of the *Bdnf* gene by a construct containing a bicistronic mRNA encoding *Bdnf* and *Gfp* separated by a short sequence designated P2A previously shown to prevent the elongation of the peptide chain (Szymczak et al., 2004). Fertile homozygote animals were generated using this construct to replace the *Bdnf* coding sequence. Brain sections of the corresponding transgenic animals revealed marked differences in the levels of GFP expression between neurons. The results are discussed in the context of a recent report describing the generation of a mouse line with the *Bdnf* gene replaced by a construct encoding a BDNF–GFP fusion protein (Leschik et al., 2019) and of RNA sequencing using single cells isolated from the mouse hippocampus (Habib et al., 2016).

## Materials and Methods

### Constructs, HEK293 cell culture, transfection, and BDNF measurements

Plasmid pCMV6-BDNF was generated by inserting a PCR fragment encoding the full-length mouse BDNF protein into the BamHI site of pCMV6 (catalog #39857, Addgene; Hofer et al., 1990). pCMV–BDNF–myc was constructed by adding one copy of a myc tag at the C terminus of WT BDNF following deletion of the last 3 aa (Matsumoto et al., 2008). To generate BDNF expression constructs containing tandem repeats of myc tags, one SbfI site was first introduced into pCMV–BDNF–myc by PCR followed by inserting multi-copies of myc tags into the SbfI site of the resultant plasmid pCMV-BDNF-myc-SbfI.

The following BDNF–GFP and P2A-SV40-NLS-GFP DNA fragments were synthesized at GeneArt (Germany):

Bdnf-Gfp (PacI, BamHI, and AscI restriction sites are underlined): ttaattaagccaccatgaccatcctgtttctgaccatggtcatcagctacttcggctgcatgaaggccgctcccatgaaggaagtgaacgtgcacggccagggcaacctggcttatcctggcgtgcggacacacggcaccctggaatctgtgaacggccctagagctggcagcagaggcctgaccacaacaagcctggccgacaccttcgagcacgtgatcgaggaactgctggacgaggaccagaaagtgcggcccaacgaggaaaaccacaaggacgccgacctgtacaccagcagagtgatgctgagcagccaggtgcccctggaaccccctctgctgttcctgctggaagagtacaagaactacctggacgccgccaacatgagcatgagagtgcggagacacagcgacccagctagaagaggcgagctgagcgtgtgcgacagcatcagcgagtgggtcacagccgccgacaagaaaaccgccgtggacatgtctggcggcaccgtgaccgtgctggaaaaggtgccagtgtccaagggccagctgaagcagtacttctacgagacaaagtgcaaccccatgggctacaccaaagagggctgcagaggcatcgacaagagacactggaacagccagtgcagaaccacccagagctacgtgcgggccctgacaatggacagcaagaaaagaatcggctggcggttcatcagaatcgacaccagctgcgtgtgcaccctgaccatcaagagaggcagaggatccggcatggtgtctaagggggaggaactgttcaccggcgtggtgcccatcctggtggaactggatggcgacgtgaacggacacaagttcagcgtgtccggcgagggcgaaggcgacgccacatacggaaagctgaccctgaagttcatctgcaccaccggcaagctgcccgtgccttggcctaccctcgtgaccacactgacctacggcgtgcagtgcttcagcagataccccgaccatatgaagcagcacgacttcttcaagagcgccatgcccgagggctacgtgcaggaaagaaccatcttctttaaggacgacggcaactacaagaccagggccgaagtgaagttcgagggcgacaccctcgtgaacagaatcgagctgaagggcatcgacttcaaagaggacggcaacatcctgggccacaagctggagtacaactacaacagccacaacgtgtacatcatggccgacaagcagaaaaacggcatcaaagtgaacttcaagatccggcacaacatcgaggacggctccgtgcagctggccgaccactaccagcagaacacccctatcggcgacggccctgtgctgctgcctgacaaccactacctgagcacccagtccgccctgagcaaggaccccaacgagaagagggaccacatggtgctgctggaattcgtgaccgccgctggcatcaccctgggcatggacgagctgtacaaatgaggcgcgcc;

P2a-Sv40_nls_-Gfp (BamHI and AscI restriction sites are underlined): ggatccggcgccaccaatttcagcctgctgaaacaggccggcgacgtggaagagaaccctggccctccaaagaagaagcggaaggtcatggtgtccaagggcgaggaactgttcaccggcgtggtgcccatcctggtggaactggatggcgacgtgaacggccacaagttcagcgtgtccggcgagggcgaaggcgacgccacctatggcaagctgacactgaagttcatctgcaccaccggcaagctgcccgtgccttggcctaccctcgtgacaaccctgacctacggcgtgcagtgcttcagcagataccccgaccacatgaagcagcacgacttcttcaagagcgccatgcccgagggctacgtgcaggaacggaccatcttctttaaggacgacggcaactacaagaccagggccgaagtgaagttcgagggcgataccctcgtgaaccggatcgagctgaagggcatcgacttcaaagaggacggcaacatcctgggccacaagctggagtacaactacaacagccacaacgtgtacatcatggccgacaagcagaaaaacggcatcaaagtgaacttcaagatcaggcacaacatcgaggacggctccgtgcagctggccgaccactaccagcagaacacccccatcggagatggccccgtgctgctgcccgacaaccactacctgagcacacagagcgccctgtccaaggaccccaacgagaagagggaccacatggtgctgctggaatttgtgaccgccgctggcatcacactgggcatggacgagctgtacaagtgaggcgcgcc.

The PacI/AscI restricted BDNF–GFP fragment was ligated into the identically restricted pAAV plasmid (Kästle et al., 2018). The BDNF-P2A-GFP expression plasmid was generated by exchanging the BamHI-AscI fragment from the before described plasmid by the BamHI-AscI gene synthesis fragment containing the teschovirus-1 P2A, the SV40 nuclear localization signal and the GFP coding sequences.

The biosynthesis and secretion of tagged BDNF proteins were analyzed using HEK293 cells transfected with plasmids encoding wild-type (WT) BDNF, BDNF–GFP, and BDNF-P2A-GFP. The enhanced version of GFP was used throughout. Cultures were maintained in Gibco DMEM supplemented with 10% FBS, 1% GlutaMAX and 1% nonessential amino acids (all Thermo Fisher Scientific). Transfections were performed in a six-well format using 2 μg of the indicated DNAs combined with 4 μl of Invitrogen Lipofectamine 2000 transfection reagent (Thermo Fisher Scientific) diluted within Gibco Opti-MEM medium (Thermo Fisher Scientific). Five hours after transfection, HEK293 cells were cultured in N2B27 medium consisting of equal volumes of Gibco Neurobasal medium and DMEM-F12 (Thermo Fisher Scientific), 1% B27 supplement (Thermo Fisher Scientific), 1% GlutaMAX, and 1% penicillin-streptomycin (Gibco Penstrep, Thermo Fisher Scientific). BSA (Sigma-Aldrich) was used at a reduced concentration of 75 μg/ml to facilitate the analysis of the conditioned media by SDS-PAGE. BDNF levels were quantified in conditioned media, and brain lysates by ELISA (Naegelin et al., 2018).

### Primary neuronal culture and transfection

Cortices of mice at embryonic day 14.5 (E14.5) for transfection and TrkB phosphorylation assays and E17.5 for immunostaining studies were collected in Hanks’ buffered salt solution (Sigma-Aldrich) and trypsinized in 1 mg/ml trypsin (Worthington) for 20 min at 37°C. The reaction was then stopped using 1 mg/ml trypsin inhibitor (Sigma-Aldrich) before the addition of 1 mg/ml DNase I (Thermo Fisher Scientific) and gentle dissociation with a 5 ml serological pipette. Cells were then pelleted by centrifugation at 1400 rpm for 5 min and resuspended in DMEM supplemented with 2% FBS, 1% GlutaMAX, and 1% Penstrep. Three hours after plating into wells coated with poly-d-lysine (Sigma-Aldrich), cells were maintained in Neurobasal medium supplemented with 1% GlutaMAX supplement, 1% Penstrep, and 2% SM1 supplement (Stem Cell Technologies). Neurons were cultured for up to 12 d with 50% media changes performed three times weekly. Subsequent transfections were performed on E14.5 neurons at 5DIV using 0.5 μg of indicated DNAs and 1 μl of Lipofectamine 2000 (see above). Depolarization of E17.5 neurons at DIV11 was achieved by supplementing media with 1 mm 4-aminopyridine (4-AP; Merck) for 24 h.

### Imaging and staining of neuronal cultures

Twenty-four hours after transfection or treatment with 4-AP, neurons were briefly washed with PBS and fixed with 4% paraformaldehyde (PFA; Thermo Fisher Scientific) for 15 min. After a 5 min permeabilization with PBS containing 0.1% Triton X-100 (PBS-T; Sigma-Aldrich), cells were incubated for 1 h in blocking solution [3% donkey serum (Sigma-Aldrich) and 1% BSA in PBS-T] at room temperature (RT). Coverslips were then incubated overnight in primary antibodies diluted in blocking solution at the following concentrations: anti-BDNF mAb #9 (7 μg/ml; Kolbeck et al., 1999), chicken anti-GFP (1:1000; catalog #ab13970, Abcam), chicken anti-MAP2 (1:5000; catalog #ab92434, Abcam), and rabbit anti-Tau (1:5000; catalog #ab64193, Abcam). Following three 5 min washes in PBS-T, cells were incubated in Invitrogen Alexa Fluor 555-conjugated anti-mouse IgG (catalog #A-31570, Thermo Fisher Scientific), Invitrogen Alexa Fluor 488-conjugated anti-chicken IgY (catalog #A-11039, Thermo Fisher Scientific), Invitrogen Alexa Fluor 647-conjugated anti-chicken IgY (Invitrogen, catalog #A-21449, Thermo Fisher Scientific), and Invitrogen Alexa Fluor 647-conjugated anti-rabbit IgG (catalog #A-21245, Thermo Fisher Scientific) secondary antibodies in blocking solution (all at 1:500 dilutions) for 1 h. After a 5 min wash with PBS-T, DAPI (Sigma-Aldrich) diluted in PBS (1:4000) was added to cells for 15 min. Coverslips were then mounted onto glass slides using Dako fluorescence mounting medium (Agilent). Images were captured using a 63× objective of a confocal microscope and are shown as maximum intensity projections of *z*-stack images (LSM 780, Carl Zeiss).

### TrkB phosphorylation assay of cultured neurons

The conditioned media of transfected HEK293 cells transfected with BDNF cDNAs were standardized to a BDNF concentration of 25 ng/ml after quantification using a BDNF ELISA (Naegelin et al., 2018). Before treatment, E14.5 neurons at 5DIV were incubated with fresh media for 15 min to aid clearance of endogenous phosphorylation. Cells were then incubated with conditioned media containing WT BDNF or BDNF-P2A for 10 min. Cells were then washed using PBS supplemented with 2 mm sodium orthovanadate (Sigma-Aldrich) to inhibit phosphatase activity and analyzed by SDS-PAGE for TrkB phosphorylation.

### Western blot and densitometric analysis

Homogenized brain tissues, HEK293 cells, and cultured neurons were incubated for 20 min on ice in RIPA buffer (50 mm Tris-HCl, 150 mm NaCl, 1 mm EDTA, 0.1% SDS, 0.2% sodium deoxycholate, and 1% Triton X-100) supplemented with phosphatase and protease inhibitor cocktail mixes, 10 μm phenanthroline monohydrate, 10 mm aminohexanoic acid, 10 μg/ml aprotonin, and 2 mm sodium orthovanadate (all Sigma-Aldrich). Lysates and conditioned media were centrifuged at 15,000 rpm to remove insoluble components before analysis by SDS-PAGE. Proteins were separated on 4–12% NuPAGE Bis-Tris gels (Invitrogen) and transferred to GE Healthcare Protran NC nitrocellulose membranes (Thermo Fisher Scientific) using a Trans-Blot semi-dry transfer unit (Bio-Rad). Membranes were subsequently blocked for 1 h in blocking solution [5% blotting-grade blocker (Bio-Rad) and 1% BSA in TBS containing 0.1% Tween (TBS-T; Sigma-Aldrich)] and then probed overnight at 4°C with antibodies to β-actin (catalog #ab8229, Abcam), BDNF (monoclonal 3C11, catalog #327-100, Icosagen), BDNF propeptide (monoclonal 5H8, catalog #sc-65514, Santa Cruz Biotechnology), GFP (catalog #ab13970, Abcam), or phosphoTrkA (Tyr674/675)/TrkB (Tyr706/707, catalog #4621, Cell Signaling Technology) in blocking solution (1:2000). Following three 10 min washes in TBS-T, membranes were incubated at RT with HRP-conjugated anti-goat (Santa Cruz, catalog #sc-2354), anti-mouse, anti-rabbit (both Promega, catalog #W4021 and W4011 respectively) or anti-chicken (Abcam, catalog #ab6877) secondary antibodies within blocking solution (1:2000). After a further three 20 min washes in TBS-T, membranes were developed using WesternBright ECL HRP Substrate (Advansta). Densitometric analysis of all blots was performed using quantification functions on Bio-Rad ImageLab software. For blots requiring BDNF quantification, recombinant BDNF standards (Regeneron/Amgen) between 300 and 18.75 pg were run alongside to create calibration curves, as appropriate.

### Animal husbandry and generation of *Bdnf-P2a-Gfp* animals

All animals in this study were approved by the Cardiff University Ethical Review Board, and all experiments were performed within the guidelines of the Home Office Animals (Scientific Procedures) Act, 1986. *Bdnf* knock-out (*Bdnf^−^* ^/^*^−^*) animals were generated by crossing mice with two floxed *Bdnf* alleles (Rauskolb et al., 2010) with mice expressing a CMV-Cre transgene (Schwenk et al., 1995). *Bdnf-P2a-Gfp* animals were generated by Taconic Biosciences. Briefly, the targeting strategy is based on NCBI transcript NM_001048139.1 and Ensemble gene ID ENSMUSG00000048482, in which exon 2 contains the complete BDNF coding sequence. The GSG sequence is then followed by the teschovirus P2A sequence (Liu et al., 2017), the SV40 nuclear localization sequence (NLS; Ray et al., 2015) and a GFP sequence inserted between the last amino acid and the translation termination codon in exon 2 of the BDNF coding sequence. The presence of the P2A sequence should result in the cotranslational generation of BDNF and NLS-GFP proteins. For the selection of positively targeted C57BL/6N Tac embryonic stem (ES) cells, a puromycin selection marker was flanked by FRT (flippase recognition target) sites and inserted into intron 1. The puromycin selection cassette was deleted in ES cells by transient expression of Flp recombinase. The remaining FRT recombination site is located in a non-conserved region of the genome and thus unlikely to interfere with BDNF expression. After blastocyst injection of targeted ES cells, chimeric animals were bred to C57BL/6N Tac mice to obtain heterozygous offspring. For colony expansion purposes, heterozygous breeding pairs were set up, with litters displaying normal Mendelian birth ratios; among 65 animals from eight litters, the distribution was as follows: wild type, *n* = 19; heterozygotes, *n* = 30; and homozygotes, *n* = 16. Animals of both sexes were used throughout the study, and the only sex-related differences were illustrated (see Fig. 3*C*). After confirming the fertility of homozygotes, the colony was then maintained using a mixture of breeding pairs. From 3 to 4 weeks of age, animals were housed in mixed genotypes and were maintained on a 12 h dark/light cycle, with access to food and water *ad libitum*.

### Tissue fixation and immunostaining

Three-month-old mice killed by pentobarbital injections were transcardially perfused with ice-cold PBS and 4% PFA, and their brains were removed and postfixed at RT for 4 h before cryoprotecting in 30% w/v sucrose solution at 4°C overnight. The following day, brains were embedded in OCT (optimal cutting temperature) compound and sectioned at 40 μm using a cryostat. Sections were blocked in blocking solution (3% donkey serum and 4% BSA in PBS-T) for 1 h before incubating overnight with mouse anti-BDNF (mAb #9) and chicken anti-GFP (1:1000). Sections were then washed three times for 10 min with PBS-T before incubating with Alexa Fluor 555 anti-mouse IgG and Alexa Fluor 488 anti-chicken IgY secondary antibodies (1:500; Thermo Fisher Scientific) for 1 h at RT. After a final wash in PBS-T for 10 min, sections were incubated with DAPI diluted in PBS (1:4000) for 20 min and mounted onto precoated polylysine slides (VWR) with Dako fluorescence mounting media. Images of gross brain regions were acquired on a confocal microscope using a 20× objective. For counts of GFP-positive nuclei, images were captured using a 63× oil-immersion and then analyzed using FIJI (Schindelin et al., 2012) and CellProfiler (McQuin et al., 2018). For each section, masks were created on FIJI to focus automated analyses onto granule cells of the dentate gyrus (DG) and pyramidal cells of CA1, CA2, and CA3. On CellProfiler, DAPI and GFP-positive nuclei were then identified using individual IdentifyPrimaryObjects modules. GFP immunostaining was measured using MeasureObjectIntensity, and identified nuclei were categorized using custom-defined bins according to their staining intensity (under categories “Below Threshold,” “Light,” “Moderate,” “Heavy,” or “Very Heavy”).

### Statistical analysis

Data were analyzed using Microsoft Excel 2013 and RStudio software. For analysis of *Bdnf-P2a-Gfp* bodyweights, a Kruskal–Wallis test was used with a Conover-Iman *post hoc* test for multiple comparisons. TrkB activation by BDNF fusion proteins was compared against that of BDNF-myc and analyzed using a one-sample *t* test. An adjusted *p* value (≤0.0125) was considered significant after a Bonferroni correction for multiple comparisons. Differences in BDNF and GFP signal intensities in depolarized *Bdnf-P2a-Gfp* neurons were analyzed using a Student’s *t* test. All results were expressed as the mean ± SE, and *p* ≤ 0.05 was considered to be significant unless otherwise stated.

## Results

### *In vitro* experiments with transfected cells

**Figure 1.**
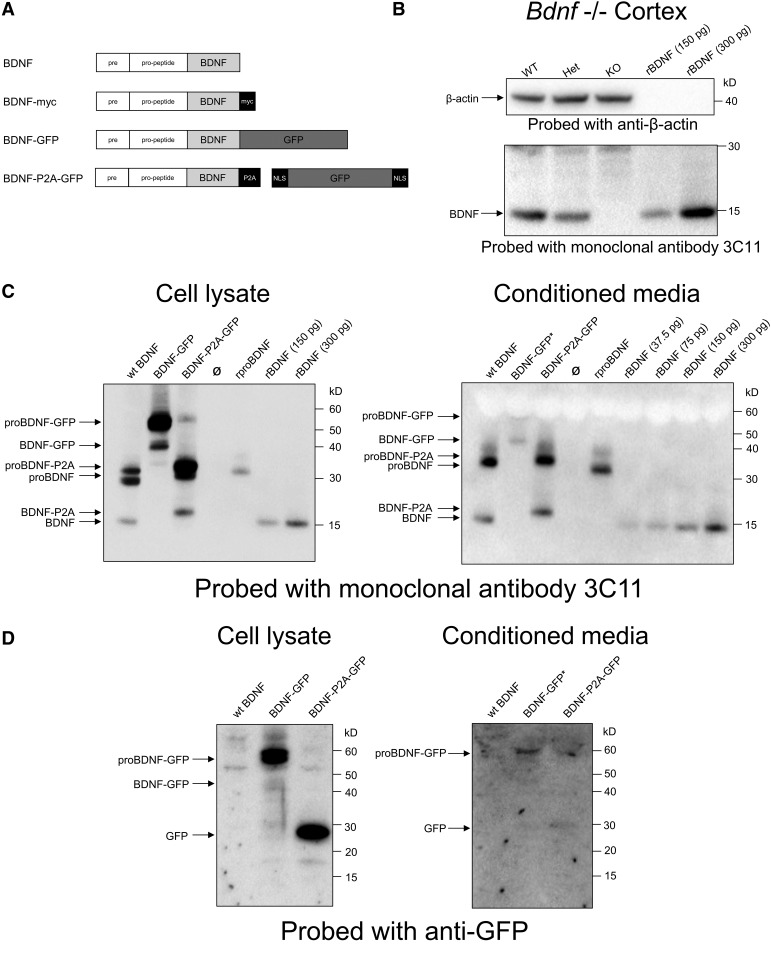
Constructs, validation of the monoclonal antibody 3C11, and transfection of HEK293 cells with BDNF expression plasmids. ***A***, Schematic representation of BDNF plasmid translation products. ***B***, Validation of monoclonal antibody 3C11 for BDNF Western blot using brain lysates from *Bdnf* WT, heterozygous (Het), and knock-out (KO) littermates at postnatal day 7. ***C***, ***D***, Western blot analysis of cell lysates and conditioned media using anti-BDNF (mAb 3C11; ***C***) and anti-GFP (***D***). Cells were transfected with the indicated plasmids. Note that three times more conditioned media was loaded into lanes for BDNF–GFP to aid detection of low levels.

As the biosynthesis and secretion of biologically active BDNF is a prerequisite for the generation of viable animals, the suitability of candidate *Bdnf* constructs was first tested using transfected HEK293 cells. Constructs encoding unmodified BDNF, BDNF directly fused with GFP or separated from BDNF by a P2A sequence (Fig. 1*A*), was introduced into expression vectors and used to transfect HEK293 cells with Lipofectamine. The GFP sequence adds 238 aa to the C terminal of the BDNF, while P2A adds 22 aa. Both cell lysates and conditioned media were collected and probed with the BDNF monoclonal antibody 3C11. This antibody unambiguously identifies BDNF in Western blot, as demonstrated by the absence of signal in lysates prepared from the cerebral cortex of *Bdnf^−^*^/^*^−^* animals (Fig. 1*B*). In transfected cells, unlike in the case with neurons expressing endogenous *Bdnf* (Matsumoto et al., 2008), a significant proportion of the immunoreactive material in cell lysates migrates as pro-BDNF identified using the pro-BDNF antibody 5H8 (data not shown) that can also be detected in the conditioned medium (Fig. 1*C*). In cell lysates of cells transfected with BDNF–GFP constructs, significant levels of pro-BDNF–GFP can be detected with the anti-BDNF antibody, while BDNF–GFP is barely detectable in the conditioned medium (Fig. 1*D*). By contrast, the bulk of pro-BDNF-P2A is clearly separated from BDNF-P2A, and both are readily detectable in the conditioned medium (Fig. 1*C*). The upward shift of BDNF-P2A compared with recombinant BDNF indicates that the P2A sequence remains attached to BDNF (Fig. 1*C*). Cell lysates probed with GFP antibodies confirm the biosynthesis of BDNF and GFP as separate products when encoded by the BDNF–P2A–GFP construct, unlike the case for the BDNF–GFP fusion construct, with the bulk of the immunoreactive material detected as unprocessed pro-BDNF–GFP (Fig. 1*D*).

**Figure 2.**
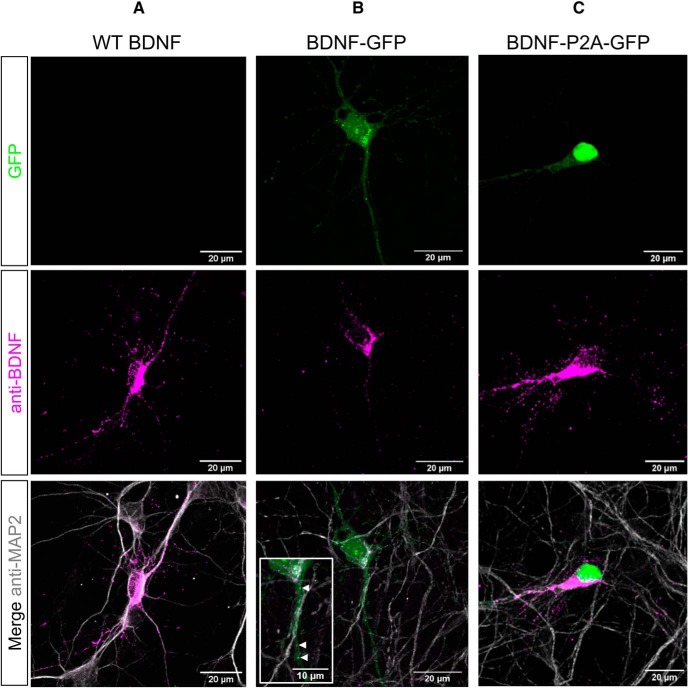
BDNF and GFP localization in transfected primary neurons. ***A–C***, E14.5 cortical cultures at 6DIV transfected with cDNAs encoding WT BDNF (***A***), BDNF–GFP (***B***), and BDNF-P2A-GFP (***C***), and stained using antibodies against BDNF and MAP2. In all transfections, the majority of BDNF immunoreactivity was observed in cell bodies in areas likely corresponding to the Golgi apparatus. In BDNF–GFP transfected cells (***B***), the separation of GFP fluorescence (green) from BDNF immunofluorescence (magenta) was observed in both the nucleus and proximal neurites (indicated by white arrowheads).

As transfected HEK293 cells do not have a dedicated secretory pathway comparable to neurons, the same three constructs were also used to transfect cultured cortical neurons (Fig. 2). The WT BDNF expression constructs revealed intense staining of neuronal cell bodies as well as dotted staining of MAP2-positive processes (Fig. 2*A*). With BDNF–GFP constructs, a GFP signal was observed throughout the transfected neurons with the GFP signal partially overlapping with the BDNF immunoreactive signal (Fig. 2*B*, arrowheads), possibly indicating that a fraction of GFP separates from BDNF (see Discussion). Neurons transfected with the BDNF–P2A constructs revealed a GFP signal largely overlapping with the nucleus, confirming the biosynthesis of BDNF and GFP as separate products in transfected neurons (Fig. 2*C*).

### Characterization of transgenic animals carrying the *Bdnf–P2a–Gfp* replacement construct

**Figure 3.**
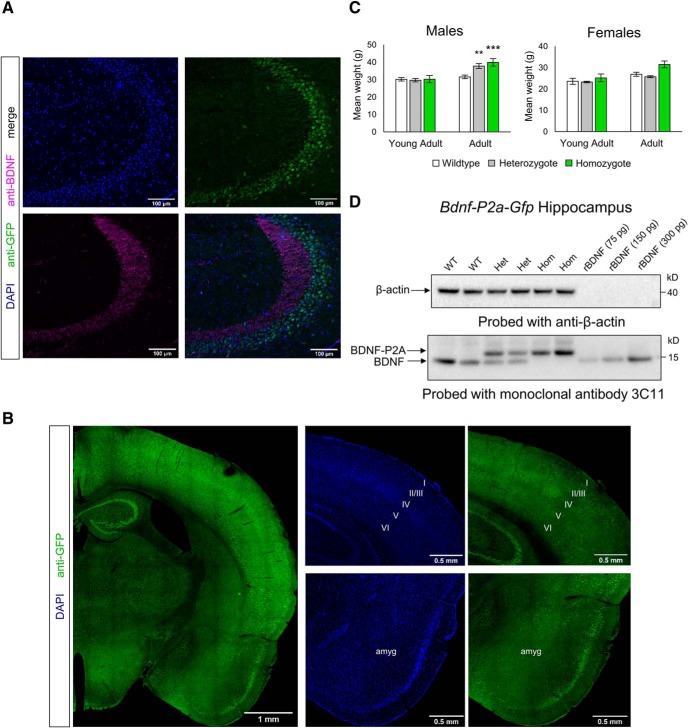
Characterization of *Bdnf-P2a-Gfp* mice. ***A***, Co-staining of BDNF and GFP homozygous *Bdnf-P2a-Gfp* hippocampus. Note the clear separation of GFP and BDNF in the mossy fiber projections of hippocampal CA3. ***B***, GFP staining of homozygote brains reveals a comparable staining pattern to previous *in situ* hybridization experiments, with staining in distinct cortical layers, hippocampal formation, and amygdala. ***C***, Body weights of young adult (3- to 4-month old) and adult (6- to 7-month old) *Bdnf-P2a-Gfp* mice. While there were no significant differences observed between littermates during young adulthood, significant weight gain could be observed in both heterozygous and homozygous males by 6–7 months of age (*p* = 0.0100 and *p =* 0.0017, respectively). The bars represent the mean weights ± SE, *n* ≥ 7 across genotypes and age categories. ***D***, Western blot analysis of adult *Bdnf-P2a-Gfp* brain lysates. Note the shift in the molecular weight of BDNF after the addition of the P2A sequence, and the separation of BDNF-P2A from GFP in *Bdnf-P2a-Gfp* heterozygous (Het) and homozygous (Hom) animals (two animals shown per genotype).

Having established the suitability of the BDNF–P2A–GFP construct with regard to the biosynthesis and secretion of BDNF as well as the biosynthesis of BDNF and GFP as distinct products, this construct was then used to replace the protein-coding region of the endogenous *Bdnf* gene. Following mating of heterozygote animals, homozygote animals carrying the *Bdnf*–*P2a*–*Gfp* construct were born at the expected Mendelian ratio (see Materials and Methods). In addition, the transgene did not measurably interfere with the fertility of the animals. Coronal brain sections of homozygous animals were then examined by confocal microscopy following perfusion, fixation, and staining with antibodies to GFP, BDNF, as well as nuclear staining with DAPI (Fig. 3*A*). The distribution of the BDNF signal is in line with previous BDNF staining experiments using BDNF antibodies (Conner et al., 1997; Yan et al., 1997; Dieni et al., 2012), while the distribution of the GFP signal corresponds to the results of previous *in situ* hybridization studies (see also Allen brain atlas, http://mouse.brain-map.org/gene/show/11850). Selective GFP labeling can be readily observed in distinct cortical layers, including layers 2, 5, and 6 as well as in distinct nuclei including the amygdala as well as all subdivisions of the hippocampal formation (Fig. 3*A*,*B*). Quantification of the GFP signal using CellProfiler (Materials and Methods) revealed that the vast majority of hippocampal neurons translate the construct at readily detectable, albeit different, levels, with the largest number of heavily labeled cells found in CA3 and the highest proportion of weakly labeled cells found in CA2 and CA1, and in the DG (Table 1). We also monitored the postnatal weight gain of the *Bdnf-P2a-Gfp* animals and observed that starting at ∼6 months, homozygote animals gained more weight than their wild-type littermates, a trend that was even visible in male heterozygotes (Fig. 3*C*). As the literature indicates that BDNF levels and TrkB signaling are critical in the regulation of food intake (Lyons et al., 1999; Kernie et al., 2000), both in mice and humans (Yeo et al., 2004), we quantified BDNF levels by ELISA in the cerebral cortex of *Bdnf-P2a-Gfp* animals and found them unchanged compared with age-matched controls: (mean ± SEM) 35.5 ± 2.11 ng/g WT cortex; and 45.4 ± 6.38 ng/g for homozygote *Bdnf-P2a-Gfp* animals. The corresponding values for the hippocampus were 97.4 ± 6.00 ng/g and 109.3 ng/g ± 17.11 for WT and homozygous animals, respectively. To confirm that GFP is cleaved after the BDNF–P2A sequence *in vivo*, we analyzed the lysates of cortices from wild-type, heterozygote, and *Bdnf-P2a-Gfp* homozygote animals by Western blot (Fig. 3*D*). These experiments revealed a quantitative upward shift of BDNF–P2A compared with the endogenous protein.

**Table 1:** Quantification of GFP signal intensity in the hippocampal formation

**Region**	**Proportions of GFP-positive cells**				
	**Background**	**Light**	**Moderate**	**Heavy**	**Very heavy**
**DG**	**5.37%**	**55.26%**	**32.08%**	**6.66%**	**0.64%**
sem	1.79%	6.33%	5.05%	2.95%	0.24%
**CA1**	**4.11%**	**65.43%**	**24.25%**	**5.39%**	**0.81%**
sem	1.33%	7.38%	5.43%	3.47%	0.69%
**CA2**	**5.52%**	**57.25%**	**26.69%**	**9.93%**	**0.61%**
sem	1.55%	7.65%	4.96%	3.90%	0.26%
**CA3**	**6.96%**	**31.50%**	**37.82%**	**17.58%**	**6.15%**
sem	0.92%	4.11%	2.05%	2.64%	1.45%

The results are based on sections from three different, 3-month-old female homozygous animals. Five sections per animal were used, and quantification was performed using CellProfiler (Materials and Methods). Quantification of the GFP signal was performed by recording the intensity of the Alexa Fluor 488 in sections stained with chicken anti-GFP primary antibody and Alexa Fluor 488-conjugated anti-chicken IgY secondary antibody. Counts were based on DAPI-stained nuclei in the DG granule cell layer and in the pyramidal cell layer for CA1/CA2/CA3. All analyzed sections fell between bregma coordinates −1.355 and −2.88.

### TrkB activation by tagged BDNF

**Figure 4.**
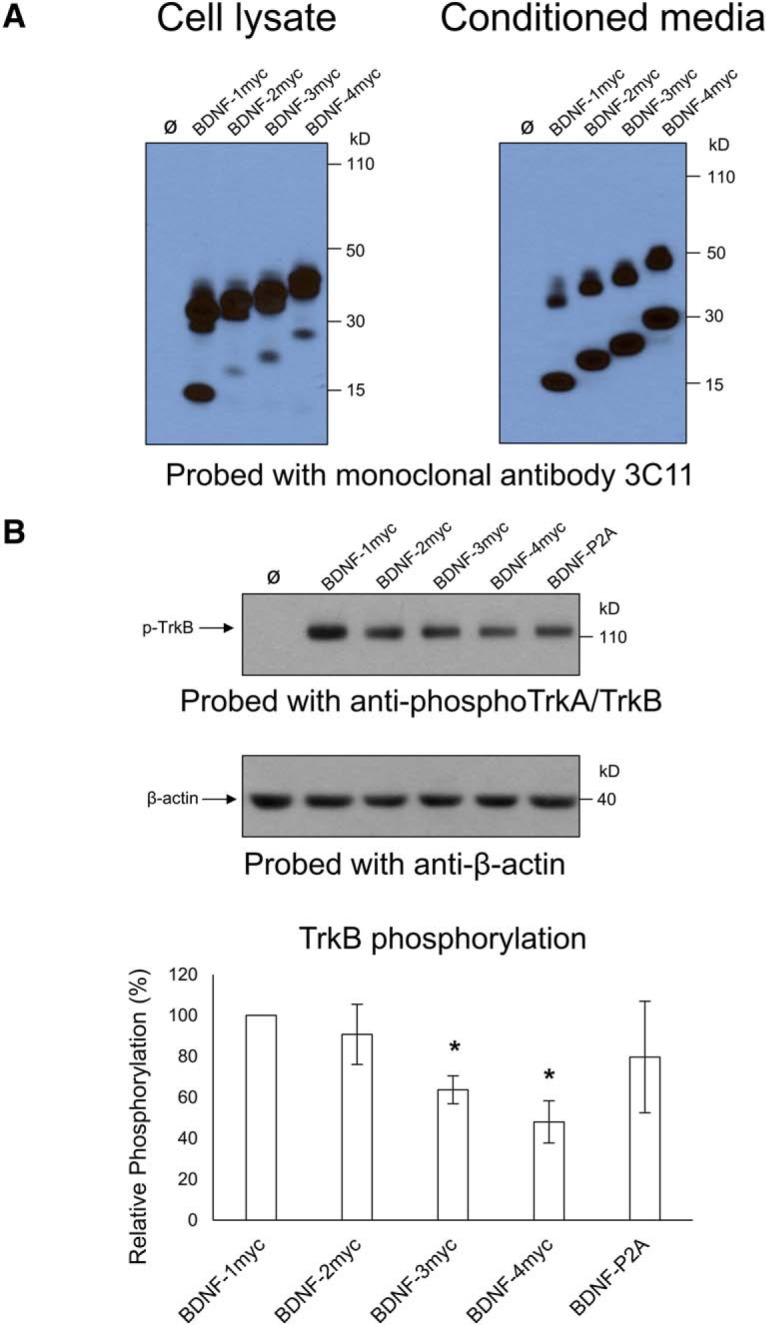
Increasing the length of BDNF-fusion proteins attenuates their ability to phosphorylate TrkB. ***A***, Western blot analysis of cell lysates and conditioned media using anti-BDNF (mAb 3C11). Cells were transfected with cDNAs encoding BDNF carrying multiple additions of the 10 aa myc tag. ***B***, TrkB phosphorylation in primary neurons treated with conditioned media containing BDNF fusion proteins standardized to 25 ng/ml. Note that the potency of TrkB phosphorylation is significantly reduced as genetically encoded tags increase in length (BDNF-3 myc, *p = 0.00312; BDNF-4 myc, *p = 0.00394). Bars representative of mean relative phosphorylation (compared with BDNF-myc) ± SE.

Given the lack of evidence for abnormal processing, levels and distribution of BDNF in cells and mice expressing the *Bdnf*–*P2a*–*Gfp* construct, we then asked whether the length of the P2A tag added to BDNF may compromise its ability to fully activate the BDNF receptor TrkB on neurons, thus conceivably explaining the abnormal weight gain of adult animals. This hypothesis was tested using BDNF constructs carrying repeats of a 10 aa myc tag used to transfect HEK293 cells. The choice of the myc tag for these experiments was inspired by previous studies indicating that the substitution of *Bdnf* by *Bdnf-Myc* allows the generation of animals with no overt phenotypes (Matsumoto et al., 2008; Dieni et al., 2012). The biosynthesis and secretion of BDNF was assessed in cell lysates and conditioned media using the BDNF antibody 3C11 and neither the biosynthesis nor the secretion of BDNF carrying up to 4 myc tags seemed to be compromised (Fig. 4*A*). The conditioned media were also used to test the ability of BDNF-myc to trigger TrkB phosphorylation (Fig. 4*B*). Primary cultures of mouse cortical neurons were exposed to HEK293 cell-conditioned media with their concentrations adjusted to correspond to 25 ng/ml BDNF as determined by ELISA. The conditioned media of HEK293 cells transfected with a BDNF–P2A–GFP construct were used in parallel (Fig. 4*B*). These experiments revealed that the ability of BDNF–3 myc and especially of BDNF–4 myc constructs to activate TrkB was reduced (Fig. 4*B*).

### Localization and quantification of BDNF and GFP in cultured neurons after depolarization

**Figure 5.**
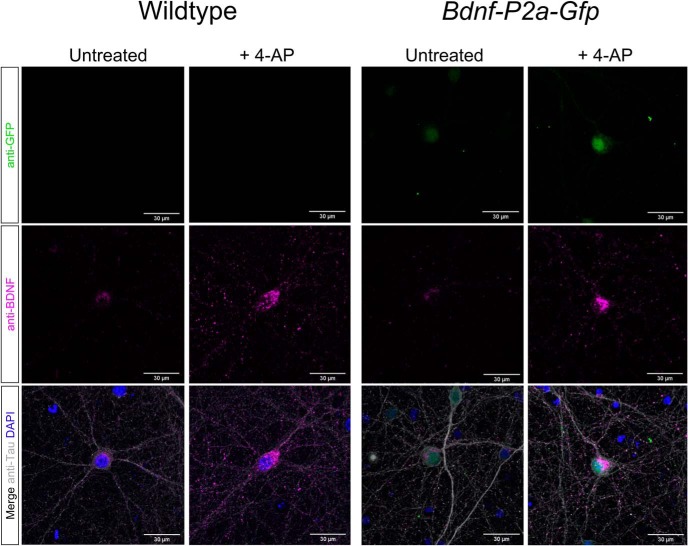
BDNF localization in wild-type versus homozygous *Bdnf-P2a-Gfp* neurons. Immunostaining of primary neurons with antibodies against BDNF (mAb #9), GFP, and Tau. After 24 h of treatment with 4-AP, note the increased number of BDNF puncta in neuronal projections and the increased GFP signal intensity in *Bdnf-P2a-Gfp* cultures. Quantification of immunostained *Bdnf-P2a-Gfp* cultures revealed significant increases in both BDNF and GFP following 4-AP treatment. Quantification of immunostained *Bdnf-P2a-Gfp* cultures revealed significant increases in both BDNF and GFP following 4-AP treatment (*p* = 3.94 × 10^−21^ and 7.85 × 10^−24^ respectively; *n* = 90 for both conditions).

Having established the localization of the BDNF signal and the segregation from the GFP signal in transfected neurons (Fig. 2*C*), it was of interest to compare these results with those obtained with neurons obtained from the *Bdnf-P2a-Gfp* mouse. As illustrated in Figure 5, the results are indistinguishable from those obtained with wild-type neurons stained with BDNF antibodies, indicating that the P2A tag does not significantly interfere with the distribution of BDNF. To test whether the intensity of the GFP signal is proportional to the BDNF immunoreactive signal, both were quantified before and after depolarization with 1 mm 4-AP. Both signals were found to increase by more than twofold after 24 h (Fig. 5).

## Discussion

The main conclusion of this study is that GFP can be used as a surrogate marker to identify cells translating the *Bdnf* mRNA in the adult mouse brain. The results also indicate that the GFP signal intensity is proportional to the degree of BDNF translation as revealed by experiments with cultured neurons. While BDNF and GFP are obviously very different proteins with different half-lives, GFP is mostly targeted to the nucleus, whereas BDNF accumulates in vesicles. This differential subcellular localization may explain why the relative signal intensities after acute depolarization do not perfectly match quantitatively (Fig. 5). In the brain, the distribution of the GFP is in remarkable agreement with the known distribution of the *Bdnf* signal observed in previous *in situ* hybridation studies, including the Allen Brain Atlas (http://mouse.brain-map.org/gene/show/11850). Importantly, the distribution of the endogenous BDNF protein remains unchanged when comparing the staining of BDNF in the hippocampal formation (Fig. 3*A*) with previous results using antibodies to myc- or hemagglutinin-tagged versions of the *Bdnf* gene (Matsumoto et al., 2008; Yang et al., 2009). This distribution is also in agreement with results obtained with rat brain sections with the then available, validated BDNF polyclonal antibodies (Conner et al., 1997; Yan et al., 1997). The fact that neither the viability of homozygote animals nor their fertility is compromised further suggests that BDNF-dependent circuits are likely to remain functional. However, the *Bdnf-P2a-Gfp* mice do abnormally gain weight several months after birth, especially in males, suggesting that these animals may be best investigated as young adults in future studies. As the BDNF levels are unchanged in these animals (see Results; Fig. 1*B*), it is conceivable that the 22 aa tag attached to the C terminal of BDNF may chronically reduce TrkB activation *in vivo*, thus potentially explaining the progressive weight gain that is apparent in male animals at ∼6 months of age. Submaximal activation of TrkB over extended periods of time *in vivo* may impair the functionality of the circuitry involved in the feeding behavior of the transgenic animals. These results also suggest that there is only limited scope to add extended tags to BDNF while fully preserving biological activity. In particular, TrkB activation with the 4 myc tag construct (adding 40 aa) is reduced by ∼50%. Caution should then be exerted when using comparatively large fusion constructs such as BDNF–GFP as they would seem unlikely to efficiently activate TrkB. The results presented in Figure 1 also indicate additional problems with the processing of pro-BDNF–GFP and the secretion of BDNF–GFP is barely detectable in the conditioned medium of HEK293 cells transfected with BDNF–GFP constructs (Fig. 1). This conclusion contrasts with the results detailed in a recent, closely related study on *Bdnf* gene substitution with GFP directly coupled to the C terminal of BDNF (Leschik et al., 2019). This gene replacement strategy led to a decrease of ∼50% of the expected Mendelian ratio of animals homozygote for the replacement of *Bdnf* by *Bdnf*-*Gfp*. In addition, the distribution of the GFP signal in these animals does not report the distribution of the endogenous BDNF protein, as exemplified by the lack of enrichment of the GFP signal in mossy fiber terminals (see above). It is conceivable that GFP may have been cleaved from BDNF in the surviving animals as a functional cleavage site at the C terminal of BDNF has been noted following the isolation of BDNF from brain homogenates (Rodríguez-Tébar et al., 1991). However, it should also be noted that this tentative explanation does not account for the Western blot results included in the study by Leschik et al. (2019).

The approach described here now opens the possibility to use the GFP signal to isolate and sort cells from the adult brain based on GFP signal intensity, thus allowing their individual profiling by RNAseq. Such results would help to inform the development of drugs selectively targeting these neurons and may deliver new clues as to the endogenous regulators of BDNF expression. Similar objectives could in principle also be reached by randomly isolating single cells from brain regions of interest without prior cell marking. As such data are indeed available for the adult mouse hippocampus (Habib et al., 2016), we compared them with those reported here. The main outcome of this comparison is that the hierarchy is somewhat different from what can be inferred from *Bdnf* mRNA levels. In particular, the study by Habib et al. (2016), indicates that the dentate gyrus contains the highest number of cells containing *Bdnf* mRNA (see *Bdnf* in https://portals.broadinstitute.org/single_cell/study/SCP1/-single-nucleus-rna-seq-of-cell-diversity-in-the-adult-mouse-hippocampus-snuc-seq#study-visualize), possibly due to the selective inclusion of DAPI-positive cells in the granule cell and pyramidal cell layer. We also note that the results summarized in Table 1 closely match previous *in situ* hybridization studies in the rat (Conner et al., 1997) and the mouse (see e.g., http://mouse.brain-map.org/gene/show/11850).

In conclusion, the mouse line reported in this study should facilitate the detailed characterization of brain neurons actively translating the *Bdnf* mRNA by allowing the selection of cells based on the intensity of the GFP signal. This should prove useful toward the development of new drugs aiming at selectively increasing the levels of BDNF in brain regions of interest, including rapidly acting depressants such as ketamine thought to act by increasing BDNF translation (Björkholm and Monteggia, 2016).

## Synthesis

Reviewing Editor: Jeffery Twiss, University of South Carolina Decisions are customarily a result of the Reviewing Editor and the peer reviewers coming together and discussing their recommendations until a consensus is reached. When revisions are invited, a fact-based synthesis statement explaining their decision and outlining what is needed to prepare a revision will be listed below. The following reviewer(s) agreed to reveal their identity: Lino Tessarollo, Tõnis Timmusk. Note: If this manuscript was transferred from JNeurosci and a decision was made to accept the manuscript without peer review, a brief statement to this effect will instead be what is listed below.

Wosnitzka et al. report the generation and characterization of a new mouse line to detect expression and translation of BDNF in brain circuitries. The authors employed a bicistronic construct encoding BDNF with a P2A sequence preceding GFP to allow detection of cells where BDNF translation into protein occurs. This is a well done study that adds a new useful tool for the identification as well as functional and anatomical evaluation of brain circuits involving BDNF signaling since the expression patterns of BDNF and GFP in this mouse line are very similar to endogenous BDNF expression pattern. The authors also demonstrate that BDNF-P2A (but not BDNF-GFP fusion protein) is efficiently secreted and able to activate BDNF receptor TrkB and that the generated mice are viable and do not differ from wild-type mice, except slight gain of weight in older mice. This mouse line will be a valuable tool for studying BDNF biology, allowing the localization of single cells in the adult mouse brain translating BDNF and studying action of drugs/stimuli believed to increase BDNF translation. The reviewers felt that the manuscript presents a thorough study and will be an important addition to the neurotrophic factor field and of general interest for the wide readership of eNeuro journal. Both reveiwers raised the minor comments outlined below, but agreed that these could be handled with review of a revised manuscript by the reviewing editor.

Using the GFP protein as a surrogate of BDNF protein expression may not be entirely precise if the proteins have different half-lives. This may be relevant for studies on temporal analysis of BDNF expression. One reviewers raised that this point should be included in the discussion and the reviewing editor agrees.

The last sentence in abstract about the comparison with other studies is not needed and should be apparent to the reader from the introduction of the paper.

In the Methods the antibodies used should be described more specifically by including not only companies but also the catalogue numbers of these antibodies.

In Methods, section “TrkB phosphorylation assay of cultured neurons”, it is written that “Cells were then incubated with conditioned media containing WT BDNF, BDNF-GFP or BDNF-P2A for 10-minute. Cells were then washed using PBS supplemented with 2 mM sodium orthovanadate (NaOV) (Sigma-Aldrich) to inhibit phosphatase activity and analysed by SDS-PAGE for TrkB phosphorylation.” However, in the Results section TrkB phosphorylation by BDNF-GFP was not studied.

Page 9 Line 382 the citation Leschik et al need to be completed/fixed her eas well as in the references section page 11 line 443-445. Line 462-the reference is also not completed.

## Author Response

Rebuttal letter, specific changes:

Comments on the Visual Abstract for Author (Required): Visual Abstract now submitted

Synthesis Statement for Author (Required):

1. Using the GFP protein as a surrogate of BDNF protein expression may not be entirely precise if the proteins have different half-lives. This may be relevant for studies on temporal analysis of BDNF expression. One reviewer raised that this point should be included in the discussion and the reviewing editor agrees. Now added at the beginning of Discussion

2. The last sentence in abstract about the comparison with other studies is not needed and should be apparent to the reader from the introduction of the paper. Removed

3. In the Methods the antibodies used should be described more specifically by including not only companies but also the catalog numbers of these antibodies. Added

4. In Methods, section “TrkB phosphorylation assay of cultured neurons”, it is written that “Cells were then incubated with conditioned media containing WT BDNF, BDNF-GFP or BDNF-P2A for 10-minute. Cells were then washed using PBS supplemented with 2 mM sodium orthovanadate (NaOV) (Sigma-Aldrich) to inhibit phosphatase activity and analysed by SDS-PAGE for TrkB phosphorylation.” However, in the Results section TrkB phosphorylation by BDNF-GFP was not studied. Corrected

5. Page 9 Line 382 the citation Leschik et al need to be completed/fixed her as well as in the references section page 11 line 443-445. Line 462-the reference is also not completed. Amended
